# A new paradigm for artesunate anticancer function: considerably enhancing the cytotoxicity via conjugating artesunate with aptamer

**DOI:** 10.1038/s41392-021-00671-8

**Published:** 2021-09-10

**Authors:** Yingying Li, Yongbo Peng, Yan Tan, Wenjing Xuan, Ting Fu, Xue-Qiang Wang, Weihong Tan

**Affiliations:** 1 Molecular Science and Biomedicine Laboratory (MBL), State Key Laboratory of Chemo/Bio-Sensing and Chemometrics, College of Chemistry and Chemical Engineering, College of Biology, Aptamer Engineering Center of Hunan Province, Hunan University, Changsha, PR China; 2 Institute of Molecular Medicine (IMM), Renji Hospital, Shanghai Jiao Tong University School of Medicine, and College of Chemistry and Chemical Engineering, Shanghai Jiao Tong University, Shanghai, PR China; 3 The Cancer Hospital of the University of Chinese Academy of Sciences (Zhejiang Cancer Hospital), Institute of Basic Medicine and Cancer (IBMC), Chinese Academy of Sciences, Hangzhou, Zhejiang China

**Keywords:** Cancer therapy, Drug delivery

**Dear Editor**,

Artemisinin and its derivatives (AIDs) have recently been widely applied in cancer therapy as promising therapeutic agents owing to its hypotoxicity and special bioactivation pathways. Though numerous strategies have been established to improve the potency of AIDs, they are still relatively inefficacious against cancers, especially as a monotherapy.^1^ It is thus essential to develop novel approaches to considerably enhance their anticancer efficacy. We hypothesized that aptamers with specific recognition, high-affinity binding, and receptor-mediated internalization would promote the accumulation of AIDs inside the target cells and lead to death of the target cancer cells.^2^

We selected sgc8c aptamer, which can specifically recognize tyrosine kinase protein 7 (PTK7) on cancer cellular membrane, to conjugate with artesunate through C-N bond formation to obtain the Sgc8c-artesunate conjugate (SAC). A random DNA sequence without targeting ability was used to prepare the control sequence-artesunate conjugate (CSAC) (Supplementary Figs. 1–14 and Tables 1–4). We selected PTK7 highly expressed CEM (Human leukemic lymphoblast cell line) and HCT116 (Human colorectal carcinoma cell line) as positive cell lines, and Ramos and HepG2 (human hepatocellular carcinoma cell line) as negative cell lines^3^ (Fig. 1a). To verify if the conjugation of the artesunate would affect the specific recognition ability of Sgc8c, flow cytometry assays were performed using Cy5-labeled Sgc8c, CSAC and SAC. The nearly same fluorescence shift of SAC and Sgc8c compared with CSAC and Sgc8c control indicated that the conjugation with artesunate did not affect the targeting ability of Sgc8c to CEM cells and HCT116 cells (Fig. 1b). In contrast, both Sgc8c and SAC could not recognize the PTK7-negative Ramos cells and K562 cells.

**Fig. 1:**
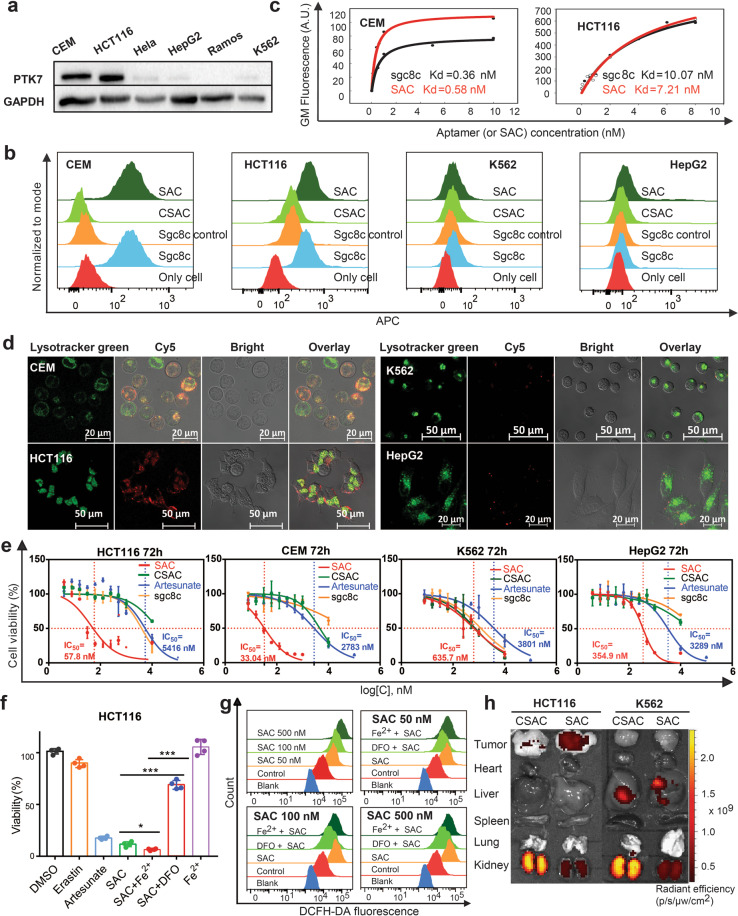
**a** Validation of PTK7 expression levels in different cancer cell lines by Western blots. **b** Flow cytometry assay of the binding of SAC, CSAC, sgc8c control and sgc8c (250 nM, Cy5-labeled sequences). **c** Flow cytometry analysis of binding affinity of SAC to PTK7-positive cell lines, CEM and HCT116. **d** Confocal microscopy shows that SAC (250 nM, Cy5-labeled) could selectively internalize into PTK7- positive CEM and HCT116 cells after 2 h incubation at 37 °C. **e** Cell viability analysis by CCK8 assay (SAC, the red curves; artesunate, the blue curves). **f** The activation of SAC is related to Fe^2+^. The addition of exogenous FeSO_4_ (10 μM) and iron chelator DFO (10 μM) were incubated with HCT116 cells for 2 h and then they were removed and SAC (10 μM) was added and incubated with HCT116 for 48 h. 10 μM DMSO, erastin and artesunate were added as control groups. ***P < 0.001, *P < 0.02; *n* = 4. **g** Measurement of the generation of reactive oxygen species (ROS) triggered by SAC (50, 100, 500 nM respectively): Cellular ROS levels were measured by dichlorofluorescein diacetate (DCFH-DA) (200 μM Fe^2+^, 200 μM DFO respectively, 5 μM DCFH-DA) after 24 h incubation with SAC. **h** The distribution of Cy5-labeled SAC (50 μM, 100 μL) and CSAC (50 μM, 100 μL) in tumor and major organs of HCT116 and K562 tumor-bearing nude mice after intravenous injection at 6 h visualized by Lumina XR in vivo imaging system

Flow cytometry also revealed that SAC retained high specificity to CEM cells and HCT116 cells with nearly the same equilibrium dissociation constant (Kd = 0.58 nM) as that of Sgc8c (Kd = 0.36 nM) (Fig. 1c). Similarly, SAC could bind to HCT116 cells with a Kd value of 7.21 nM, which was slightly lower than that of Sgc8 (Kd = 10.07 nM). These findings suggested that the SAC possesses excellent binding affinity.

The internalization ability of SAC was next examined by confocal microscopy. As presented in Fig. 1d, Cy5-labeled SAC could specifically recognize and internalize into the PTK7-positive CEM and HCT116 cell lines, as indicated by much brighter intracellular red fluorescence signals. In contrast, weak fluorescence could be detected in K562 and HepG2 cells. Besides, the merge of the lysotracker green channel with SAC channel showed colocalization outcomes both for CEM and HCT116 cells, as displayed in the overlay column. These observations demonstrated that specific recognition, high-affinity binding, and receptor-mediated internalization triggered the selective uptake of SAC into target cancer cell lines, which might be critical for achieving the enhanced cytotoxic effect of artesunate. The results of cytotoxicity studies were summarized in Fig. 1e. SAC exhibited high cytotoxicity to CEM and HCT116 cell lines compared with K562 and HepG2 cell lines. SAC showed impressive IC_50_ of 57.8 nM against HCT116 cells and 33.04 nM against CEM cells, improved 93 times and 84 times than the IC_50_ of artesunate. The control groups of artesunate, Sgc8c, and CSAC were much less efficient than SAC. Particularly noteworthy, SAC exhibited much lower cytotoxicity to K562 and HepG2 cells. These results demonstrated the importance of specific recognition of Sgc8c against its target PTK7. The increased cytotoxicity of SAC against K562 and HepG2 cells might be explained by the improved water solubility of artesunate after conjugating with aptamer and nonspecific uptake of SAC by the control cells.

It has been demonstrated that Fe^2+^ could activate the artesunate, leading to the formation of highly reactive carbon-centered radicals from AIDs, thus can trigger the alkylation of proteins and generate reactive oxygen species (ROS) to cause cancer cells death.^4^ We compared the viability of HCT116 cells when they were separately treated with same concentration (10 μM of artesunate, SAC and SAC with pre-treatment of different additives. As shown in Fig. 1f, SAC (13%) presented superior cytotoxicity compared to artesunate (17%), signifying the importance of active transporting ability. We also observed that SAC could induce more cell apoptosis in FeSO_4_ pretreated group than the artesunate alone. However, the addition of iron chelator DFO significantly reduced the cytotoxicity of SAC.^5^ These outcomes indicated that cellular uptake of Fe^2+^ could efficiently activate artesunate, thus enhancing the cytotoxicity of SAC.

We next evaluated the ROS level in the HCT116 cancer cell line after SAC treatment. As presented in Fig. 1h, the addition of SAC, irrespective of concentration, could trigger the generation and accumulation of cellular ROS. The addition of Fe^2+^ chelator DFO suppressed the ROS generation process, and the ROS generation was boosted again when additional Fe^2+^ was added. These results suggested that SAC indeed triggered the generation of ROS which might be associated with the uptake of Fe^2+^ ions. Flow cytometry-based apoptosis determination assays were carried out to verify whether SAC could induce more target cell death after 48 h incubation. As indicated in Supplementary Figs. 15, 16, and Table 5, the percentages of early apoptotic cells and late apoptotic cells increased along with the varied SAC concentration from 10 nM to 1 μM, suggesting that SAC might kill HCT116 cells through ROS-related apoptosis.

After demonstrating the aptamer enabled in vitro targeted delivery of artesunate, we wondered if SAC could retain its recognition ability and selectively reach the tumor site in vivo. PTK7-positive HCT116 cell line and PTK7-negative K562 cell line were chosen to establish tumor-xenografted mouse models. SAC was rapidly accumulated at tumor site of HCT116 bearing mice at 30 min after intravenous injection (Supplementary Fig. 17), and it could remain in the tumor for at least 6 h. Moreover, the ex vivo image of SAC-treated HCT116 tumor displayed much higher fluorescence intensity 10 h post-injection. In contrast, the fluorescence of CSAC disappeared at 3 h after intravenous injection and its ex vivo imaging showed slight fluorescence at the tumor site and high fluorescence in the kidney after 10 h post-injection (Fig. 1h and Supplementary Figs. 18, 19, and Table 6). As for K562 tumor-xenografted mice, both SAC and CSAC treated groups showed disappeared fluorescence signal at 3 h after intravenous injection. These findings demonstrated the remarkable in vivo recognition and binding abilities of SAC, thus showing the potential of specific in vivo drug delivery for efficient targeted cancer therapy.

In conclusion, SAC respectively inhibit the proliferation of HCT116 and CEM cells 93 and 84 times more than artesunate alone. Mechanistic studies revealed that aptamers with PTK7-specific recognition, high-affinity binding and receptor-mediated internalization promoted the accumulation of artesunate inside the target cells and triggered Fe^2+^-dependent artesunate bioactivation that led to the generation of alkyl radicals and reactive oxygen species, and inhibited the growth of target cells via apoptosis. Moreover, in vivo fluorescent imaging of tumor-bearing mice indicated SAC could target and remain at the tumor site for much longer time relative to control groups, further highlighting the potential of exploring artesunate as a component of an efficient targeted cancer treatment approach.

## Supplementary information

Supplementary Data

## Data Availability

All supporting data are included in the manuscript and Supplemental files. Additional data are available upon reasonable request to the corresponding author.
