# Supplementary material for: A new paradigm for artesunate anticancer function: considerably enhancing the cytotoxicity via conjugating artesunate with aptamer
Source: Signal Transduct Target Ther. 2021 Sep 10;6:327. doi: 10.1038/s41392-021-00671-8 (PMC8429419; doi:10.1038/s41392-021-00671-8)
Supplement: Supplementary file 1 — Supplementary Data [file 41392_2021_671_MOESM1_ESM.docx]

**Supplementary Data**

**A New Paradigm for Artesunate Anticancer Function: Considerably Enhancing the Cytotoxicity via Conjugating Artesunate with Aptamer**

Yingying Li^1^, Yongbo Peng^1^, Yan Tan^1^, Wenjing Xuan^1^, Ting Fu^3^, Xue-Qiang Wang*^1^, Weihong Tan*^1,2,3^

^1^Molecular Science and Biomedicine Laboratory (MBL), State Key Laboratory of Chemo/Bio-Sensing and Chemometrics, College of Chemistry and Chemical Engineering, College of Biology, Aptamer Engineering Center of Hunan Province, Hunan University, Changsha, 410082, P. R. China.

^2^Institute of Molecular Medicine (IMM), Renji Hospital, Shanghai Jiao Tong University School of Medicine, and College of Chemistry and Chemical Engineering, Shanghai Jiao Tong University, Shanghai, 200240, P. R. China.

^3^The Cancer Hospital of the University of Chinese Academy of Sciences (Zhejiang Cancer Hospital), Institute of Basic Medicine and Cancer (IBMC), Chinese Academy of Sciences, Hangzhou, Zhejiang 310022, China

**Table of contents**

**1. Materials and methods** 2

**2. Synthesis and Characterization of sequences conjugated with artesunate** 2

**Supplementary Table 1** DNA sequences used in this work……………………………..……3

**Supplementary Table 2** The elution program of HPLC 3

**Supplementary Fig. 1** Synthesis route of Sgc8c-artesunate conjugate(SAC). 3

**Supplementary Fig. 2** HPLC retention time of SAC. 4

**Supplementary Fig. 3** HPLC retention time of SAC-Cy5. 4

**Supplementary Fig. 4** Synthesis route of sgc8c control -artesunate conjugate(CSAC). 4

**Supplementary Fig. 5** HPLC retention time of CSAC. 4

**Supplementary Fig. 6** HPLC retention time of CSAC-Cy5. 5

**Supplementary Table 3** Mass data of the Sgc8c and its conjugates 5

**Supplementary Fig. 7** ESI-MS analysis of Sgc8c (5’ C6 NH_2_). 6

**Supplementary Fig. 8** ESI-MS analysis of Sgc8c (5’ C6 NH_2_, 3’ Cy5). 7

**Supplementary Fig. 9** ESI-MS analysis of SAC. 7

**Supplementary Fig. 10** ESI-MS analysis of SAC-Cy5. 8

**Supplementary Table 4** Mass data of the control sgc8c conjugate artesunate 8

**Supplementary Fig. 11** ESI-MS analysis of Sgc8c control (5’-NH_2_). 9

**Supplementary Fig. 12** ESI-MS analysis of Sgc8c control (5’ C6 NH_2_, 3’ Cy5). 9

**Supplementary Fig. 13** ESI-MS analysis of CSAC. 9

**Supplementary Fig. 14** ESI-MS analysis of CSAC-Cy5. 10

**3. Results and Discussion** 10

**Supplementary Fig. 15** Flow Cytometry image of the apoptosis caused by SAC. 10

**Supplementary Table 5** Statistics of apoptosis percentage. 10

**Supplementary Fig. 16** Analysis of the apoptosis 11

**Supplementary Fig. 17** *In vivo* images of HCT116 and K562 tumor-bearing nude mice. 11

**Supplementary Fig. 18** Fluorescence intensity in different organs of HCT116 and K562 tumor-bearing nude mice.. 12

**Supplementary Table 6** Statistics of the radiant efficiency of *ex vivo* fluorescence images of major organs and tumors. 12

**Supplementary Fig. 19** The statistic of fluorescence intensity in tumors of the HCT116 and K562 tumor-bearing nude mice in Supplementary Table 5. 12

1. **Materials and methods**
   1. **Materials.** All oligonucleotide sequences were purchased from Hippo Biotechnology Co., Ltd. Artesunate was purchased from Bidepharm Co., Ltd. (China). Ultrapure deionized water obtained from a Milli-Q Biocel system was used in all experiments.

**1.2 Instrumentation.** All products were purified through HPLC (High Performance Liquid Chromatography). Cell binding, and cellular ROS detection were performed on the Cytek Dxp Athena. Images of fluorescence characterization were acquired by confocal fluorescence microscopy (Zeiss LSM710). A VIS Lumina XR was used to capture the images of major organs and tumors of treated mice.

**1.3 Cell culture.** All the cell lines used in the experiment were purchased from China Center for Type Culture Collection (CCTCC, China). The cells were maintained in RPMI-1640 medium (or DMEM medium), supplemented with 10% fetal bovine serum, 1% penicillin-streptomycin, and incubated at 37 °C with 5% CO2 and 95% humidity. The cell lines were negative for mycoplasma.

**1.4 Targeting ability and binding affinity.** HCT116 cells and HepG2 cells were attached cells and were digested by 0.2% EDTA, then washed twice by DPBS to remove EDTA and incubated with 250 nM Cy5-labled sequences in the binding buffer on ice for 1h. Suspension cells (CEM, K562), were collected at a density of 1 x 10^5^ cells per tube, washed twice by pre-cooled DPBS and incubated with 250 nM Cy5-labled sequences (gradient concentration for binding affinity assay) on ice for 1h in the binding buffer, then washed by washing buffer to remove unbound sequences and re-suspended in 400 μL DPBS. The fluorescence intensity was detected by Cytek Dxp Athena by counting 10000 events. Cells without any drug treatment were used as control to measure background signals. Dates were analyzed by Origin Pro 8.0. Centrifugation speed for all cells are 1000 r.p.m., 3min. The equation of Ligand Binding is f = Bmax*abs(x)/(Kd + abs(x)), R=0.99.

**1.5 Confocal imaging.** HCT116 cells and HepG2 cells were seeded in a glass bottom confocal dished at a density reasonable density overnight and then incubated with 250 nM Cy5-labled samples at 37 °C with 5% CO2 and 95% humidity for 2 hours, 100 nM lysotracker green for 1 hour and then washed twice by DPBS, suspended by 500 μL DPBS and all cellular fluorescent images were collected on the FV500-IX81 confocal microscope with 60x oil immersion objective. Excitation wavelength and emission filters were as following: alexa488, 488 nm laser line excitation; alexa633, 633 nm laser line excitation. As for suspension cells(CEM, K562), 400 μL volume of cell suspension was dropped on the 35 mm glass bottom dishes then waiting for 3 min to make the cells settle down before the imaging. All the images were analyzed using the analysis software Zen 2.3.

**1.6 Cell viability.** Cell viability was evaluated by CCK8 assay. Attached cells (HCT116 and HepG2) were seeded at 96-well plates and incubated overnight in RPMI-1640 medium with 10% FBS in a final volume of 100 μL overnight. Then the drug with increasing concentration was added and incubated for 72 hours. Suspension cells (CEM, K562) were seeded at 96-well plates and incubated with drugs of gradient concentration for 72 h. After 72h incubation, 10% CCK8 assay was added and absorbance was measured at 450 nm. IC50 value were calculated by GraphPad Pro Prism 7.0 (GraphPad, San Diego, CA).

**1.7 The activation of SAC is related to Fe^2+^.** Cells were plated in the 96-well plate overnight. DMSO (10% in culture medium)-treated cells were used as control, and SAC of 10 μM was added to the well in final volume of 100 μL. The plate were incubated for 48 hours at 37 °C . Absorbance was measured at 450 nm. IC50 value were calculated by GraphPad Pro Prism 7.0 (GraphPad, San Diego, CA).

**1.8 Cell apoptosis.** Cell apoptosis analysis was performed by following the instruction of the Apoptosis and Necrosis Assay Kit (Beyotime). Brieﬂy, HCT116 cells (5×105 per well) were seeded in 24-well plates and incubated with 10 nM, 100 nM and 1 μM ART and SAC for 48 h. Then cells were washed twice, resuspended in 800 μL 1 × Propidium Iodide and Hoechst Staining Solution, then incubated at room temperature for 30 min. Finally, DNA content was measured by flow cytometry (Cytek Dxp Athena). Apoptosis was analyzed using FlowJo 7.6 software.

**1.9 Measurement of reactive oxygen species(ROS).** ROS levels in cells were measured by using dichlorofluorescein diacetate(DCFH-DA).100000 HCT116 cells per well were plated in 6-well plates and cultured for 24 hours. Cells were incubated with Cy5-labled SAC for 48 hours, then washed with DPBS twice and incubated with 5 μM DCFH-DA for 25min. Cells were then washed and harvested, fluorescence intensity was quantified by flow cytometry by counting 10000 events. Dates were analyzed by Flowjo software.

**2.0 The *ex vivo* image fluorescent images**. The image of major organs and tumors obtained from the HCT116 and k562 tumor-bearing nude mice were taken at different time point after intravenous injection with 50 μM, 100 μL cy5-labed SAC and CSAC. The *ex vivo* fluorescence images of major organs and tumors obtained from the HCT116 and K562 tumor-bearing nude mice were analyzed by VIS Lumina XR.

**2. Synthesis and Characterization of sequences conjugated with artesunate**

| Sequence Name | Sequence（5' to 3'） |
| --- | --- |
| Sgc8c | *ATCTAACTGCTGCGCCGCCGGGAAAATACTGTACGGTTAGA |
| Sgc8c control | *ATCTAACTGATTATTATTATTATTATTATTATTCGGTTAGA |
| Sgc8c-Cy5 | *ATCTAACTGCTGCGCCGCCGGGAAAATACTGTACGGTTAGA-Cy5 |
| Sgc8c control-Cy5 | *ATCTAACTGATTATTATTATTATTATTATTATTCGGTT-Cy5 |

**2.1 Sequence information.**

**Supplementary Table 1** DNA sequences used in this work.

*C6-NH**_2_** was tagged at the 5’ end of the sequence.

All the DNA sequences used for the artesunate conjugation in this manuscript are in the Supplementary table 1, and the sequence of sgc8c and its control sequence were reported before^1,2^.

**2.2 Synthesis routes.** Artesunate, N,N-dicychlohexylcarbodiimide (DCC) and N-Hydroxysuccinimide (NHS) were dissolved in DMSO and then reacted at room temperature for 1 hour. The DNA sequences were dissolved in phosphate buffer, added to the organic part, and left to react overnight. The same procedure and methods were used for control sequences conjugated with artesunate.

**2.3 HPLC Purification.** The reaction solution was purified through HPLC to get the pure product. The mixture was directly purified by reversed-phase HPLC using a BioBasic18 column. The two kinds of products termed SAC and CSAC, respectively.

| Time/min | A (0.1 M TEAA) | B (acetonitrile) |
| --- | --- | --- |
| 0 | 95% | 5% |
| 4 | 95% | 5% |
| 4.01 | 90% | 10% |
| 40 | 40% | 60% |
| 50 | 10% | 90% |

**Supplementary Table 2** The elution program of HPLC.

**Supplementary Fig. 1** Synthesis route of Sgc8c-artesunate conjugate(SAC).

**
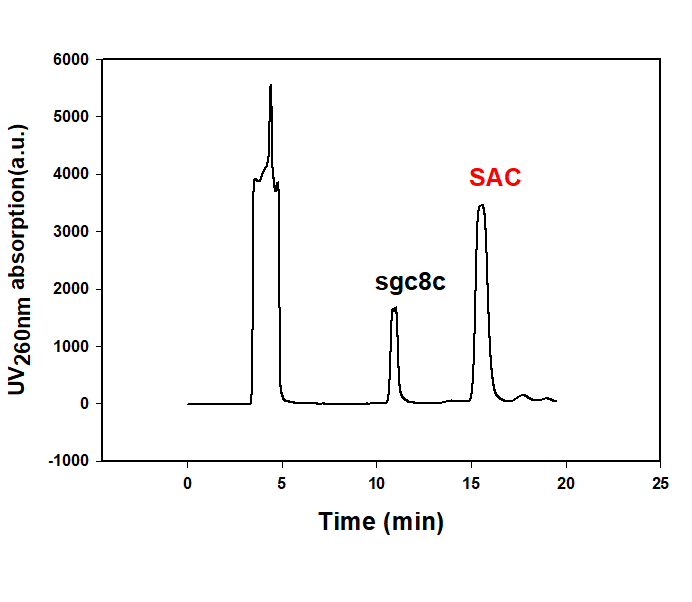
**

**Supplementary Fig. 2** HPLC retention time of SAC.

Because the artesunate-aptamer conjugate has greater hydrophobicity, it induces longer elution time (16 minutes) compared to Sgc8c aptamer (12 minutes).


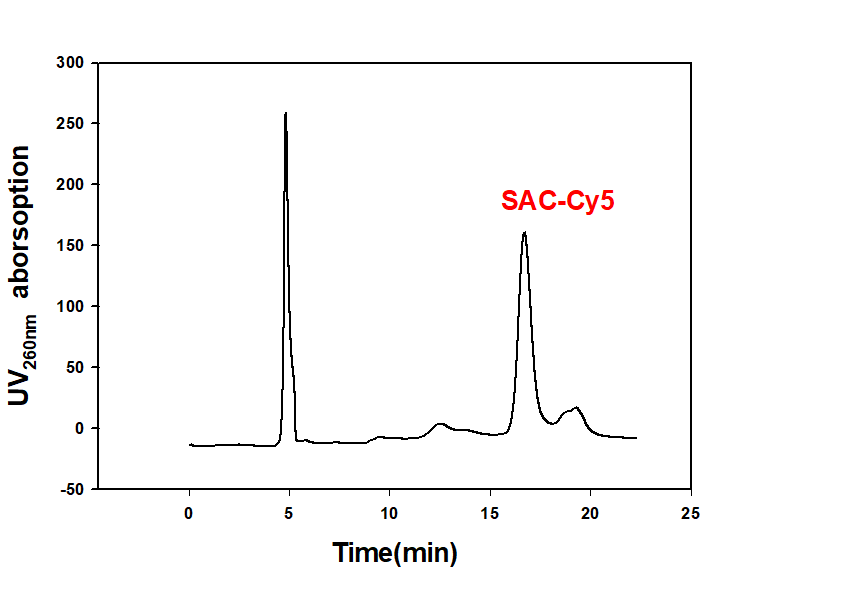


**Supplementary Fig. 3** HPLC retention time of SAC-Cy5.


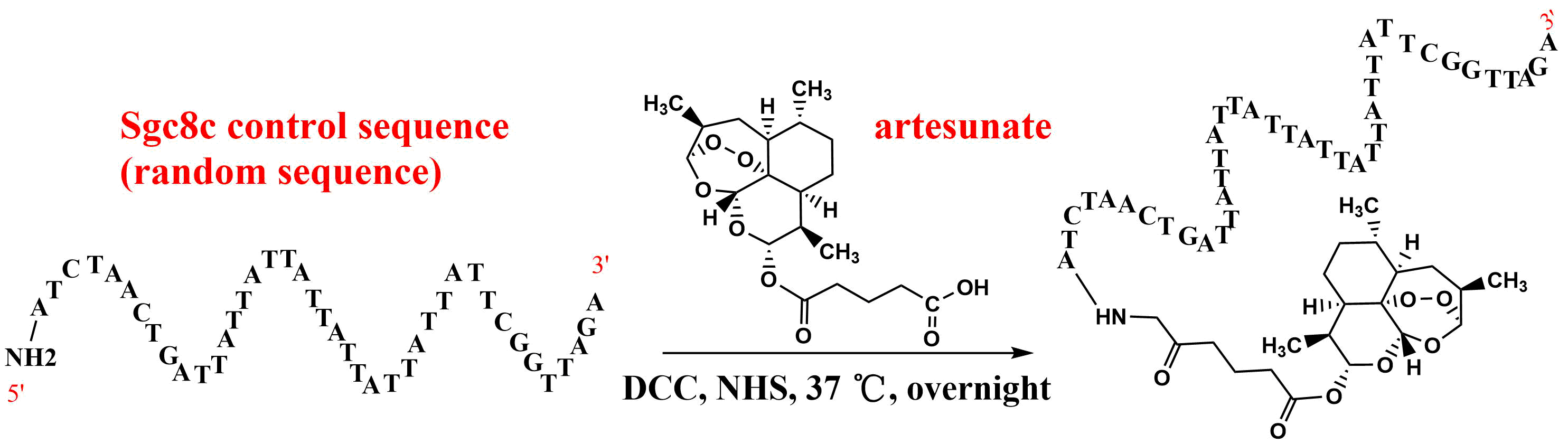


**Supplementary Fig. 4** Synthesis route of sgc8c control -artesunate conjugate(CSAC).

**
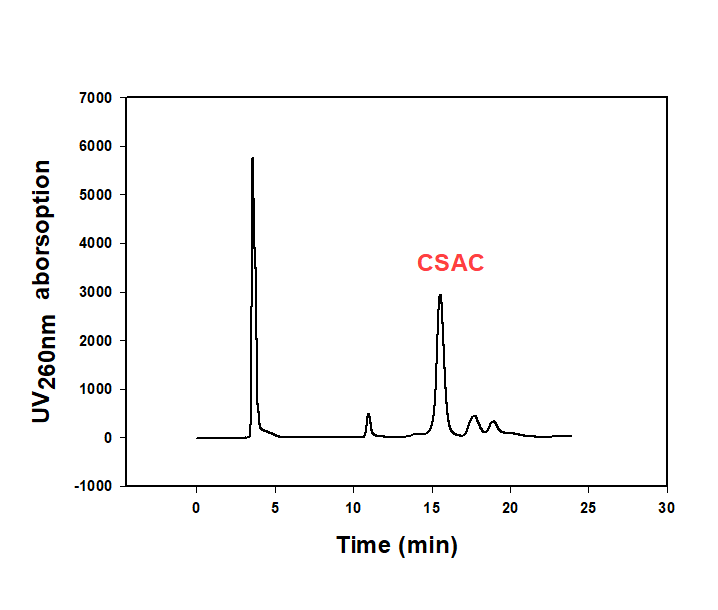
**

**Supplementary Fig. 5** HPLC retention time of CSAC.


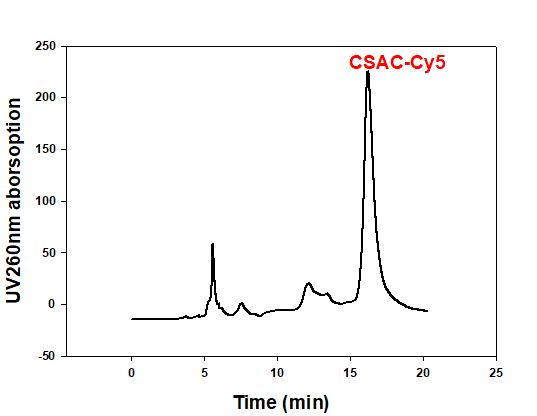


**Supplementary Fig. 6** HPLC retention time of CSAC-Cy5.

**2.4 ESI-MS analysis.**

| Only sequence | M.W | Conjugate with ART | M.W |
| --- | --- | --- | --- |
| Sgc8c (5’ C6 NH_2_) | 12811.5 | SAC | 13180.7 |
| Cy5-sgc8c (5’ C6 NH_2_, 3’ Cy5) | 13375.8 | SAC-Cy5 | 13743.7 |

**Supplementary Table 3** Mass data of the Sgc8c and its conjugates
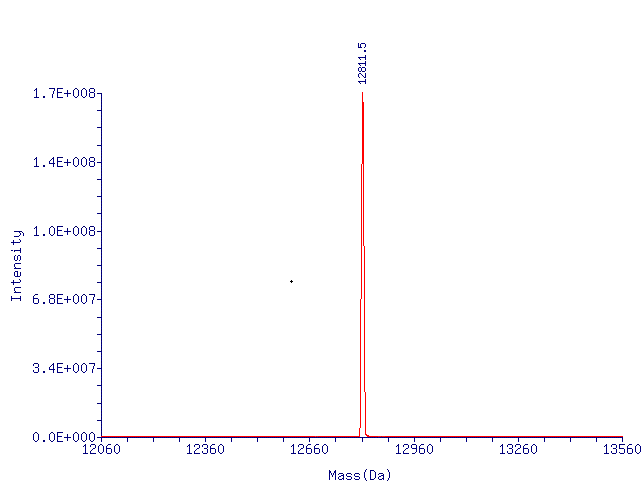


**Supplementary Fig. 7** ESI-MS analysis of Sgc8c (5’ C6 NH_2_) by Sangon (Shanghai). Observed DNA peak was 12811.5.


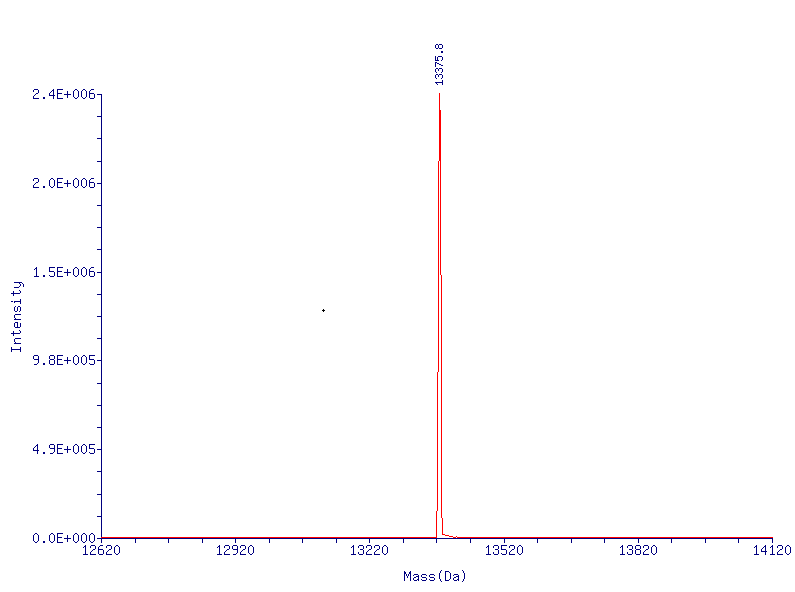


**Supplementary Fig. 8** ESI-MS analysis of Sgc8c (5’ C6 NH_2_, 3’ Cy5) by Sangon (Shanghai). Observed DNA peak was 13375.8.


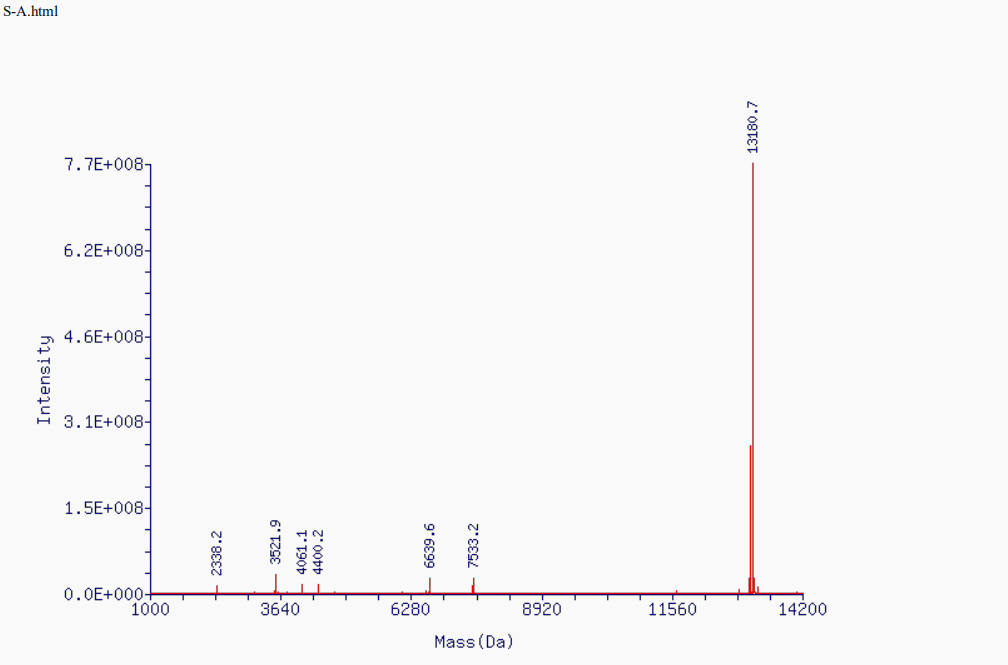


**Supplementary Fig. 9** ESI-MS analysis of SAC by Sangon (Shanghai). Observed DNA peak was 13180.7.


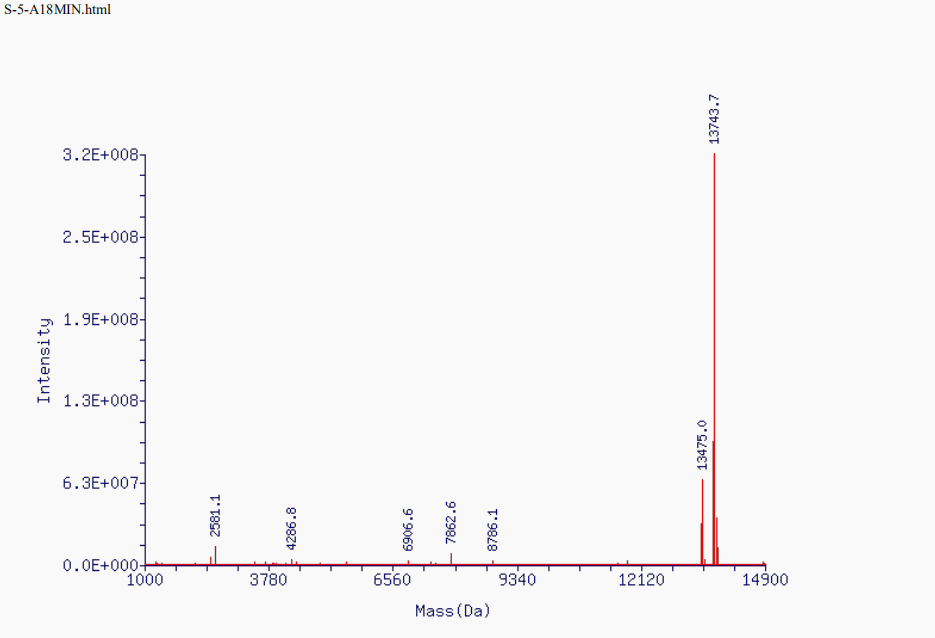


**Supplementary Fig. 10** ESI-MS analysis of SAC-Cy5 by Sangon (Shanghai). Observed DNA peak was 13743.7.

| Only sequence | M.W | Conjugate with ART | M.W |
| --- | --- | --- | --- |
| Sgc8c control (5’ C6 NH_2_) | 12762.6 | CSAC | 13128.4 |
| Cy5-Sgc8c control (5’ C6 NH_2_, 3’ Cy5) | 13323.4 | CSAC-Cy5 | 13691.9 |

**Supplementary Table 4** Mass data of the control sgc8c conjugate artesunate.


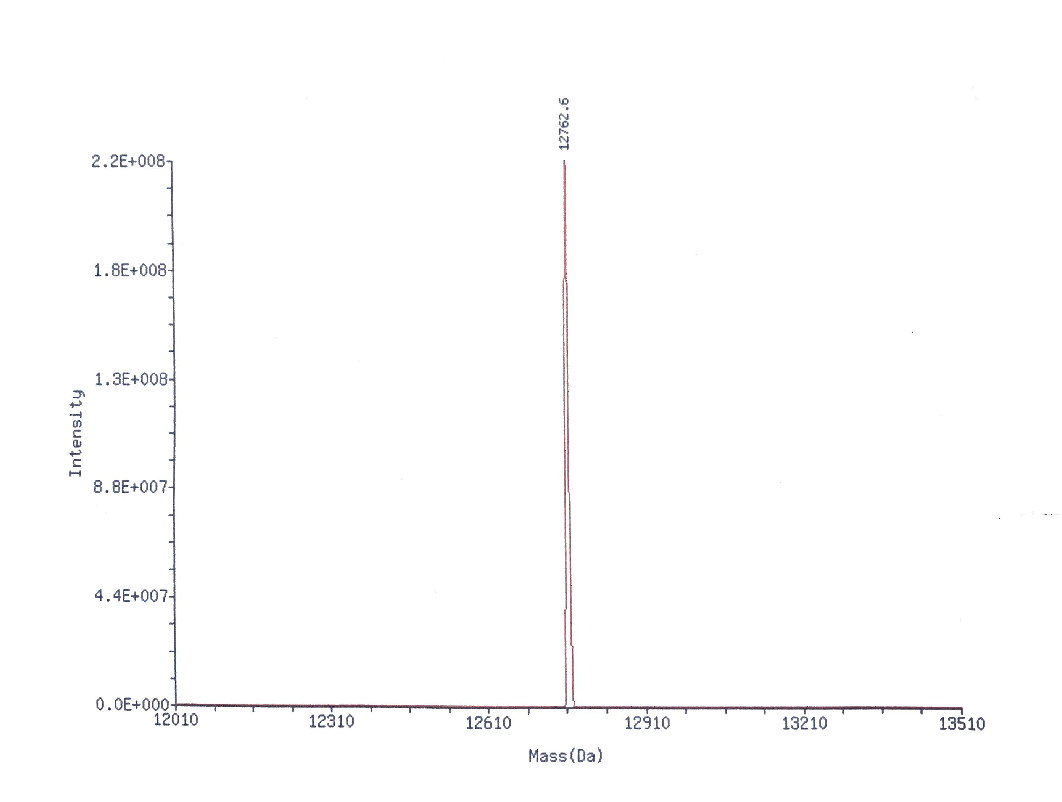


**Supplementary Fig. 11** ESI-MS analysis of Sgc8c control (5’-NH_2_) by Sangon (Shanghai). Observed DNA peak was 13128.4.


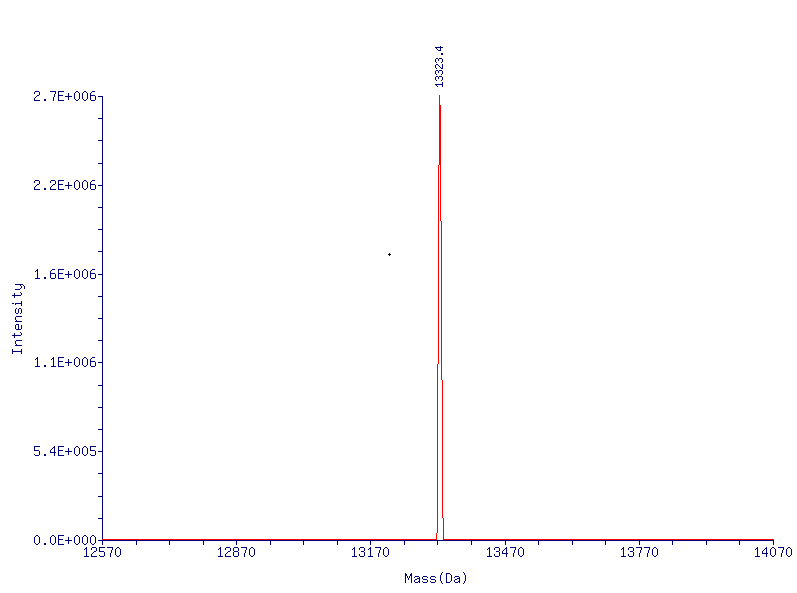


**Supplementary Fig. 12** ESI-MS analysis of Sgc8c control (5’ C6 NH_2_, 3’ Cy5) by Sangon (Shanghai). Observed DNA peak was 13323.4.


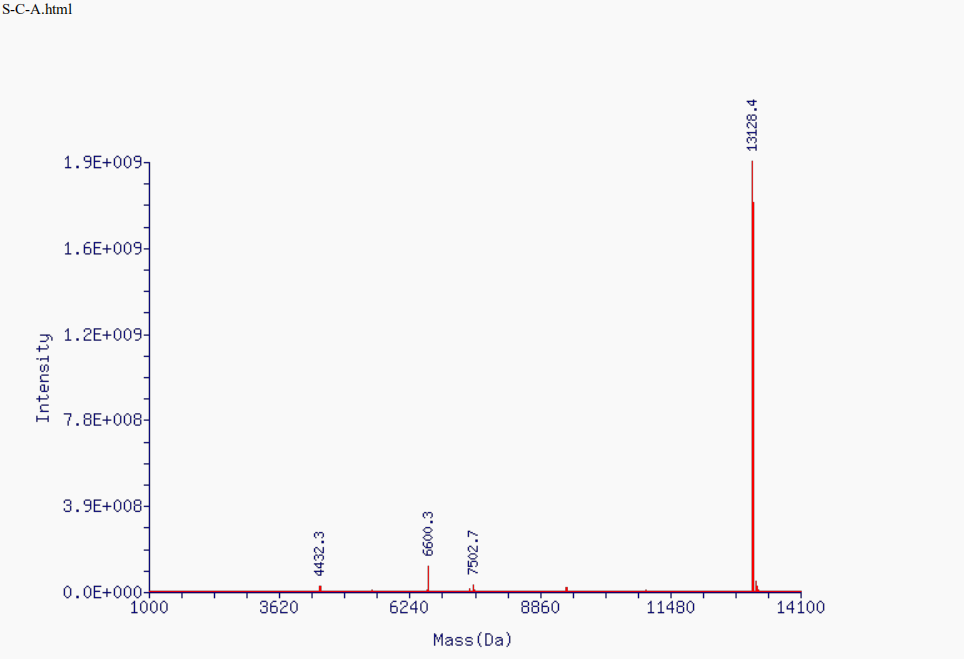


**Supplementary Fig. 13** ESI-MS analysis of CSAC by Sangon (Shanghai). Observed DNA peak was 13128.4.


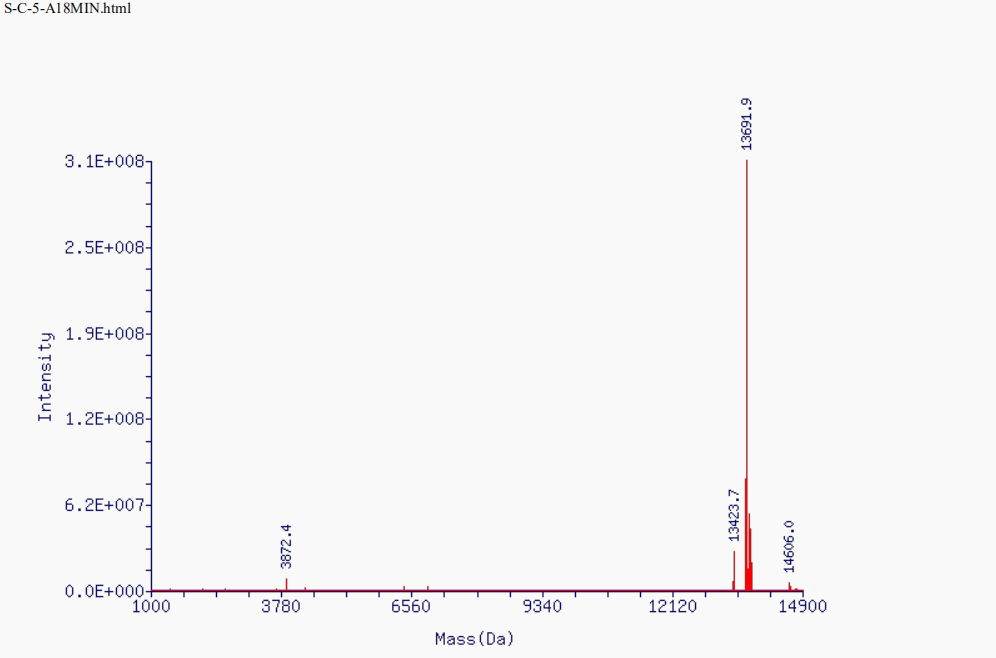


**Supplementary Fig. 14** ESI-MS analysis of CSAC-Cy5 by Sangon (Shanghai). Observed DNA peak was 13691.9.

1. **Results and Discussion**
   1. **SAC induces cell apoptosis of HCT116 cells.**


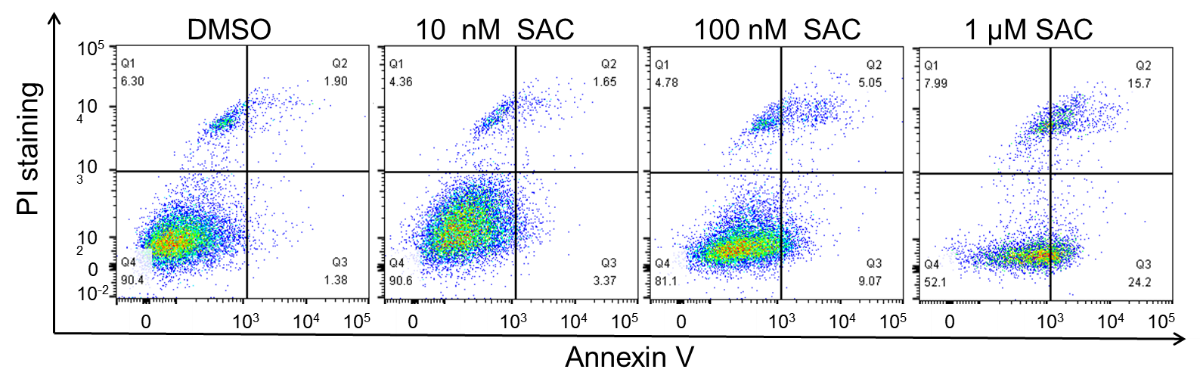


**Supplementary Fig. 15** Flow Cytometry image of the apoptosis caused by SAC.

**Supplementary Table 5** Statistics of apoptosis percentage

| Average (%) | DMSO | SAC(10 nM) | SAC(100 nM) | SAC(1 μM) |
| --- | --- | --- | --- | --- |
| Q2 | 3.686667 | 1.326667 | 4.426667 | 16.47667 |
| Q3 | 2.816667 | 2.328333 | 8.636667 | 20.607 |
| Q2+Q3 | 6.503333 | 3.655 | 13.06333 | 37.08367 |

**Supplementary Fig. 16** Analysis of the apoptosis.

**2.3 The Fluorescence Intensity of Major Organs**


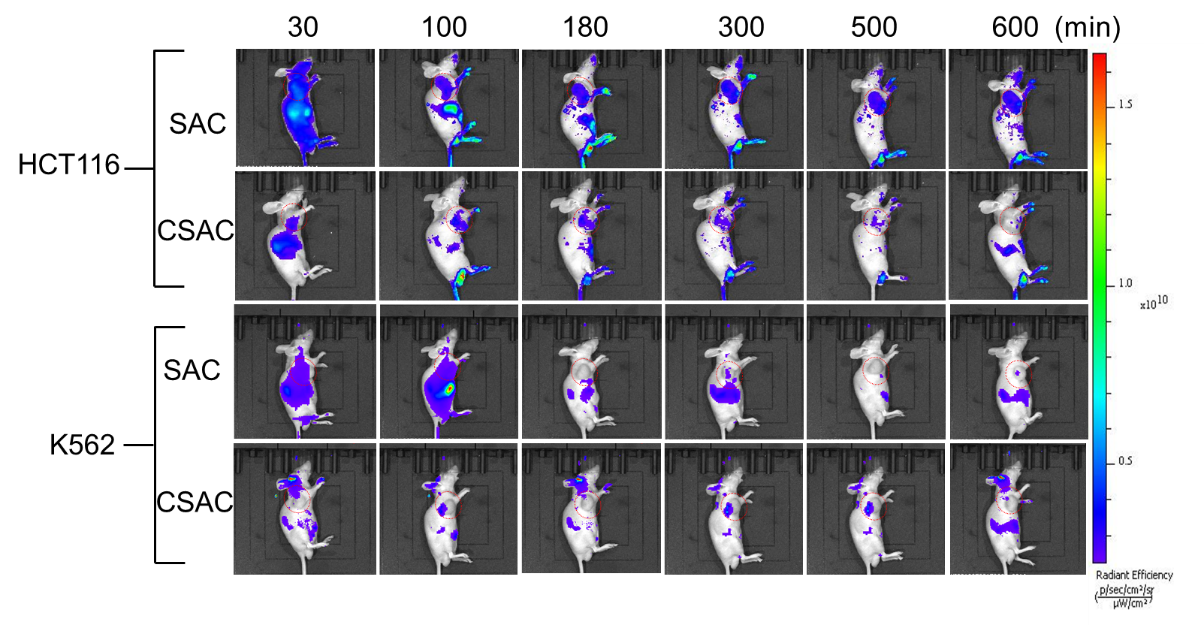


**Supplementary Fig. 17** *In vivo* images of HCT116 and K562 tumor-bearing nude mice after intravenous administration of Cy5-labeled SAC (50 µM, 100 µL) and Cy5-labeled CSAC (50 µM, 100 µL) at 30, 100, 180, 300, 500, and 600 min post-injection.


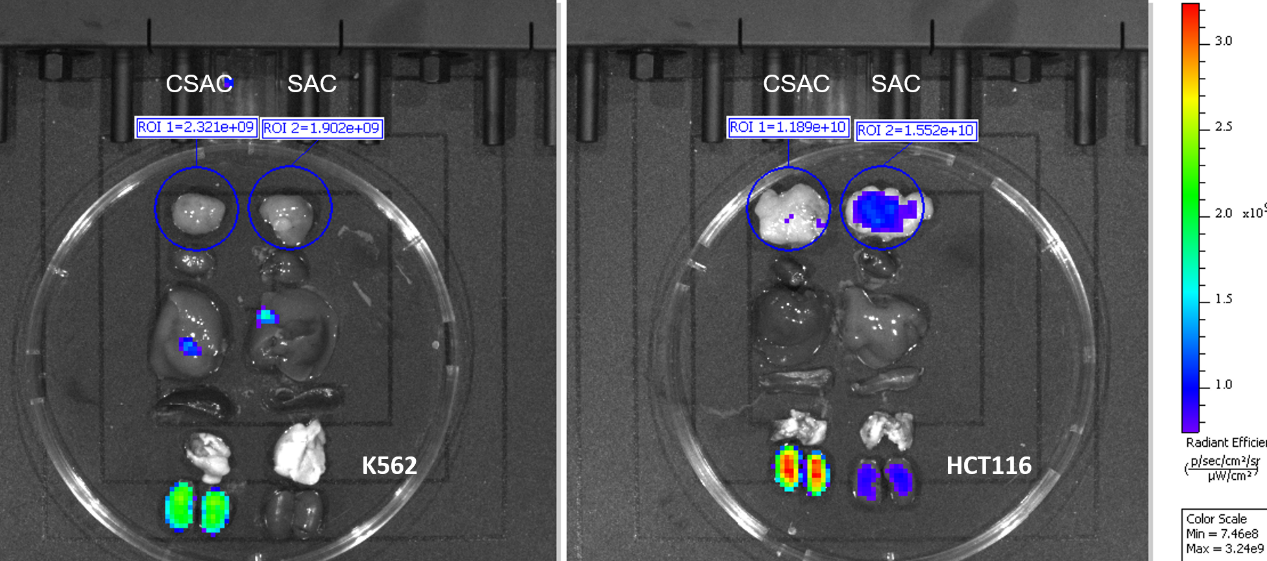


**Supplementary Fig. 18** Fluorescence intensity in different organs of HCT116 and K562 tumor-bearing nude mice.

| Image Number | HCT116-CSAC | | HCT116-SAC | | K562-CSAC | | K562-SAC | |  |  |
| --- | --- | --- | --- | --- | --- | --- | --- | --- | --- | --- |
| ROI | ROI 1 | | ROI 2 | | ROI 1 | | ROI 2 | |  |  |
| Image Layer | Overlay | | Overlay | | Overlay | | Overlay | |  |  |
| Total Radiant Efficiency [p/s] / [uW/cm^2^] | 11900000000 | | 15500000000 | | 2320000000 | | 1900000000 | |  |  |
| Avg Radiant Efficiency [p/s/cm^2^/s] / uW/cm^2^] | 374000000 | | 494000000 | | 73100000 | | 60600000 | |  |  |
| Stdev Radiant Efficiency | | 281000000 | | 410000000 | | 98600000 | | 74900000 | | |
| Min Radiant Efficiency | | 7900000 | | 6380000 | | 2580000 | | 2050000 | |  |
| Max Radiant Efficiency | | 799000000 | | 1210000000 | | 280000000 | | 257000000 | |  |

**Supplementary Table 6** Statistics of the radiant efficiency of *ex vivo* fluorescence images of major organs and tumors obtained from the HCT116 and K562 tumor-bearing nude mice.

**Supplementary Fig. 19** Statistic of fluorescence intensity in tumors of the HCT116 and K562 tumor-bearing nude mice in Supplementary Table 6.

1 Dihua Shangguan, Y. L., Zhiwen Tang, Zehui Charles Cao, Hui William Chen, Prabodhika Mallikaratchy, Kwame Sefah, Chaoyong James Yang, and Weihong Tan. Aptamers evolved from live cells as effective molecular probes for cancer study. *PNAS* **103**, 11838–11843 (2006).

2 Shangguan, D., Tang, Z., Mallikaratchy, P., Xiao, Z. & Tan, W. Optimization and modifications of aptamers selected from live cancer cell lines. *Chembiochem* **8**, 603-606 (2007).
